# Reduced Function and Diversity of T Cell Repertoire and Distinct Clinical Course in Patients With *IL7RA* Mutation

**DOI:** 10.3389/fimmu.2019.01672

**Published:** 2019-07-17

**Authors:** Atar Lev, Amos J. Simon, Ortal Barel, Eran Eyal, Efrat Glick-Saar, Omri Nayshool, Ohad Birk, Tali Stauber, Amit Hochberg, Arnon Broides, Shlomo Almashanu, Ayal Hendel, Yu Nee Lee, Raz Somech

**Affiliations:** ^1^The National Lab for Diagnosing SCID - The Israeli Newborn Screening Program, Pediatric Department A and the Immunology Service, Jeffrey Modell Foundation Center, Sheba Medical Center, Edmond and Lily Safra Children's Hospital, Israel Ministry of Health, Tel HaShomer, Israel; ^2^The Mina and Everard Goodman Faculty of Life Sciences, Advanced Materials and Nanotechnology Institute, Bar-Ilan University, Ramat-Gan, Israel; ^3^Sheba Cancer Research Center and Institute of Hematology, Sheba Medical Center, Tel HaShomer, Israel; ^4^Sackler Faculty of Medicine, Tel Aviv University, Tel Aviv, Israel; ^5^The Wohl Institute for Translational Medicine, Sheba Medical Center, Tel HaShomer, Israel; ^6^Soroka Medical Center, Genetics Institute, The National Institute for Biotechnology in the Negev, Ben-Gurion University of the Negev, Beer Sheva, Israel; ^7^Department of Pediatrics, Hillel Yaffe Medical Center, Hadera, Israel; ^8^Faculty of Health Sciences, Soroka University Medical Center, Pediatric Immunology Clinic, Ben-Gurion University of the Negev, Beer Sheva, Israel; ^9^The National Center for Newborn Screening, Israel Ministry of Health, Tel HaShomer, Israel

**Keywords:** IIL7Rα, PID, SCID, NBS, TREC, immune repertoire

## Abstract

The alpha subunit of IL-7 receptor (IL7R7α) is critical for the differentiation of T cells, specifically for the development and maintenance of γδT cells. Mutations in *IL7RA* are associated with Severe Combined Immunodeficiency (SCID). Infants with *IL7RA* deficiency can be identified through newborn screening program. We aimed at defining the immunological and genetic parameters that are directly affected by the *IL7RA* mutation on the immune system of five unrelated patients which were identified by our newborn screening program for SCID. The patients were found to have a novel identical homozygote mutation in *IL7RA* (n.c.120 C>G; p.F40L). Both surface expression of IL7Rα and functionality of IL-7 signaling were impaired in patients compared to controls. Structural modeling demonstrated instability of the protein structure due to the mutation. Lastly the *TRG* immune repertoire of the patients showed reduced diversity, increased clonality and differential CDR3 characteristics. Interestingly, the patients displayed significant different clinical outcome with two displaying severe clinical picture of immunodeficiency and three had spontaneous recovery. Our data supports that the presented *IL7RA* mutation affects the IL-7 signaling and shaping of the *TRG* repertoire, reinforcing the role of *IL7RA* in the immune system, while non-genetic factors may exist that attribute to the ultimate clinical presentation and disease progression.

## Key Points

- Patients with a novel *IL7RA* mutation showed reduced expression and function of IL7Rα and restricted diversity of the TRG repertoire.
- The patients that initially were identified by newborn screening for SCID eventually developed different clinical course.

## Introduction

The Interleukine-7 receptor subunit alpha (IL7Rα) also known as CD127, together with IL-2Rγ (the common γ chain) subunit forms a heterodimer receptor for Interleukin-7 (IL-7). The engagement of IL-7 with its receptor activates signal transduction via the JAK1 and JAK3 tyrosine kinases, which leads to dimerization of *STAT3, STAT5A*, and *STAT5B* transcription factors, activation of the PI3 kinase pathway and up-regulation of Bcl-2 (1, 2). IL7Rα, which is expressed in lymphoid progenitors and therefore is important for IL-7 signaling, is subsequently involved in several processes, including cell survival and proliferation during lymphoid development (3) and in generation and maintenance of thymus-derived γδT cells (4, 5). In humans, IL-7 signaling plays a critical role in the development of αβT cells (6, 7), while in mice it is reported to be involved in the development of both T and B cells. Furthermore, it was shown that the IL7Rα signals are involved in the VDJ recombination process both in T and B cells (8, 9), where the combinatorial joining of the V, D, and J segments encodes for the variable regions of the T cell receptor (TCR) in T cells and B cell receptor (BCR a.k.a. Immunoglobulins) in B cells. This allows for the generation of the diversity and plasticity of the adaptive immune system which is critical for the development of T and B cells. Specifically, since TCRγ chain undergoes rearrangement prior to the TCRβ chain, TCRγ (*TRG)* genes rearrangement are directly affected by IL-7 signaling, as was shown in IL7Rα^−/−^ mice that harbor severe impairment in the γ locus rearrangement (4, 10–12). However, direct effect of IL-7 signaling on the rearrangement of other loci (i.e., TCRβ, TCRα, TCRδ) is still unknown. Altogether, IL7Rα delivers trophic signals that protect lymphoid progenitors from a death process and maintain the viability of cells during gene rearrangement (7, 13–15).

Not surprisingly, patients with *IL7RA* mutations present with Severe combined immunodeficiency (SCID). SCID is a fatal primary immunodeficiency (PID) that is caused by mutations in genes critical in the development of T, B, and natural killer (NK) cells. Classification of SCID is traditionally based on the presence or absent of these cells. Typical SCID is defined when the affected infant has <300 (autologous) CD3^+^ T cells/μl, proliferation to PHA is severely reduced and other supporting features such as detectable maternal T cells in peripheral blood and/or proven deleterious defect in a known SCID gene. Leaky SCID is defined when the patient has 300–1,500 CD3^+^ T cells/μl, few naive T cells, moderately reduced proliferation rate to PHA and incomplete defect in a known SCID gene (16). Patients with mutations in the *IL7RA* gene are typically characterized by T^−^B^+^NK^+^ immune phenotype. Similar to other SCID phenotypes, patients with IL7Rα deficiency are predisposed to acquired opportunistic infections early in life, displaying with poor outcome and even death, unless their immune system is restored by means of hematopoietic stem cell transplantation (HSCT). Currently, SCID patients can be identified immediately after birth using the T cell receptor excision circles (TREC)—based newborn screening assay (16). This screening program has been successfully implemented in the past several years, in different countries, including Israel (17), enabling early diagnosis and prompt treatment to affected infants. While most patients with *IL7RA* mutations have a severe form of T cell immunodeficiency (18), some have a partial deficiency (19), with residual cells, leaky phenotype (20), and delayed age of onset (21). Nevertheless, characterization of such patients has established a major role for deciphering the IL-7-receptor-dependent signaling in T cell development in humans (22).

Here we report, five un-related infants who were identified by the Israeli national newborn screening (NBS) program for SCID and subsequently were found to have identical *IL7RA* mutation. They all underwent thorough immune and genetic investigations. Despite having identical *IL7RA* mutation, with initial T cell lymphopenia, the patients ultimately displayed diverse clinical and immunological course, resulting in different treatment approach and outcome: two patients displayed severe clinical picture of immunodeficiency (one required HSCT and one unfortunately died) and three had spontaneous recovery without clinical manifestation of immunodeficiency. The variable clinical expression observed in our patients points to a possible “leakiness” of this specific mutation. Nevertheless, in this study, the role of IL-7 signaling in T cell proliferation and VDJ recombination process in humans is reinforced, specifically at the *TRG* locus.

## Methods

### Patients

The patients were identified via the Israeli NBS program and their diagnosis was validated at the national laboratory at the “Edmond and Lily Safra” Children's Hospital. The Institutional Review Board (Sheba Medical Center, Tel HaShomer) approved this study and a written informed consent was obtained from their parents according to the ethical declaration of the Helsinki committee.

### Immunological Evaluation

Cell surface markers of peripheral blood mononuclear cells (PBMCs), lymphocyte proliferative response to mitogens, and the amount of T-cell receptor excision circles (TRECs) were determined as previously described (23). The analysis of T cell receptor (TCR) Vβ expression were determined according to manufacturer's manual (Beta Mark TCR Vβ Repertoire Kit, Beckman Coulter).

### Whole Exome and Sanger Sequencing

High throughput sequencing for whole exome sequencing was performed on genomic DNA samples from patients, coding regions were enriched with a SureSelect Human All Exon V5 Kit (Agilent) and then sequenced as 100-bp paired-end runs on an Illumina HiSeq 2500 (Illumina Inc).

We used the BWA mem algorithm (version 0.7.12) (24) for alignment of the sequence reads to the human reference genome (hg19). The HaplotypeCaller algorithm of GATK version 3.4 was applied for variant calling, as recommended in the best practice pipeline (25). KGG-seq v.08 was used for annotation of identified variants (26) and in house scripts were applied for filtering, based on family pedigree and local dataset of variants detected in previous sequencing projects. The *IL7RA* mutation was validated by dideoxy Sanger sequencing in the patients and carriers. Data were evaluated using Sequencer v5.0 software (Gene Codes Corporation).

### Measurements of IL7Rα (CD127) Expression

Fresh whole blood was used to stain for surface IL7Rα expression using αCD3-FITC (clone UCHT1, Beckman coulter) and αCD127-BV421 (clone A019D5, BioLegend) antibodies, followed by measurement and analysis using fluorescence-activated cell sorting (FACS, NAVIOS, Beckman Coulter) and the Kaluza software (Beckman Coulter).

### Stimulation and Expression of Intracellular pSTAT5

Whole blood samples were incubated with either IL-7 (1 ng/ml) or IL-2 (1,000 ng/ml) (PeproTech) at 37°C in a water bath for 15 min. Measurements of p-STAT5 expression in CD4^+^ T cell subsets were performed according to manufacturer's protocol (PerFix EXPOSE, Beckman Coulter). Staining was performed using αCD45-KO (clone J33), αCD4-APC (clone 13B8.2), and αPhospho-STAT5(Tyr694)-PE (clone C71E5) antibodies (Beckman Coulter). The measurement and analysis were carried out using FACS (NAVIOS, Beckman Coulter) and the Kaluza software (Beckman Coulter).

### Computational Structure Modeling of *IL7RA* Mutation

Structural analysis of IL7Rα was based on PDB (24) structures for isolated IL7R (3UP1) and for the IL7Rα/IL-7 complex (3DI2). Molecular graphic images were created using Jmol and Chimera (25). Contact analysis of the wildtype and mutated residues were conducted using G23D (26) which apply the SCCOMP program (27) for side chain modeling. Functional predictions of the variant were conducted using Polyphen2 (28), Sift2 (29), and MutationTaster (30). Thermostability prediction were conducted using Imutant2 (31), Cupsat (32), Maestro-web (33), and SDM (34).

### TRG Immune Repertoire Sequencing by NGS

TCR library was generated from patients' and controls' genomic DNA using primers for conserved regions of V and J genes in the *TRG* (T cell receptor Gamma) loci, according to manufacturer's protocol (LymphoTrack, Invivoscribe Technologies). Quantified libraries were pooled and sequenced using Mi-Seq Illumnia technology (Illumina Inc). The sequences were subjected to bioinformatics analyses to generate FASTA sequence files, which then were submitted to the IMGT HighV-QUEST webserver (http://www.imgt.org) and analyzed further for Hierarchical Treemap (Macrofocus Gmbh), Shannon's H, and Simpson's D diversity indices and frequency of the different gene usages. Shannon's H and Simpson's D were calculated using the following equations:

$$\begin{array}{lll} \text{Shanno}{\text{n}}^{\text{′}}\text{sH} & = & -\sum_{i=1}^{R}\;p_{i}\text{ In }p_{i}\\ \text{Simpso}{\text{n}}^{\text{′}}\text{sD} & = & \sum_{i=1}^{R}\;p_{i}^{2} \end{array}$$

*D* = Dominance, unevenness

*R* = Total templates

*i* = Unique rearrangements

p_*i*_ = Proportion of the total sequences belonging to the “i”th unique rearrangement

For raw data, please contact yuneeya4u@gmail.com.

### Statistical Analysis

Statistical analyses for one tail *t*-tests and F-tests were carried out using the Prism5 (GraphPad). For all the statistical analyses, Gaussian distribution was assumed.

## Results

### Clinical and Immunological Characterization of the Patients

Five un-related infants, with same ethnicity, all born to consanguine Muslim parents (1st degree cousins), without family history of immunodeficiency, were screened positive for SCID via the Israeli NBS program (Table 1). Thus, at two time points during the first week of life, patients showed undetectable TRECs levels on dried blood spots (normal levels is more than 36 copies per 1.5 mm card punch) and according to our NBS algorithm were referred for further confirmatory tests. In all, no evidence of secondary cause for immunodeficiency was identified. All patients were found to have lymphopenia with low T cell numbers, normal B and NK cells, undetectable or significantly reduced TREC copies in fresh blood and low response to mitogenic stimulation (Table 2). In addition, Pt1 and Pt2 were assessed for cell chimerism upon diagnosis, due to suspected trans placental-acquired maternal T cells, which is a pathognomonic feature of SCID (35). While Pt1 showed no chimerism after birth, patient 2 showed 10% chimerism confirming a diagnosis of SCID (Supplementary Figure S1).

**Table 1:** Family and clinical description.

	**Pt1**	**Pt2**	**Pt3**	**Pt4**	**Pt5**
Gestational age (weeks)	39 + 2/7	40	37	35	29 + 3/7
Birth weight (grams)	2,950	3,380	2,800	2,300	1,330
Parental consanguinity	Yes	Yes	Yes	Yes	Yes
Family history of immunodeficiency	No	No	No	No	No
Child order	5/5	1/3	1/3	1/2 (twins)	2/2
Age of first abnormal TREC results (days)	4	2	3	3	7
Clinical course	Chronic diarrhea, FTT, adenovirus, CMV viremia chest infection bacterial tracheitis	Neonatal fever, negative work-up, psoriatic-like rash at age 18 months—recovered	Normal	1 viral infection, complete recovery	Hepato-splenomegaly, diffuse erythroderma (Omenn) sepsis NEC totalis Laparotomy, short bowel resection, *E. coli* sepsis, candida infection
Past treatment	TMP+SMX/Flu IVIG, BMT at age 8 months	TMP+SMX/Flu until age 12 months	TMP+SMX/Flu until age 12 months	None	TMP+SMX/Flu IVIG broad spectrum antibiotics
Current treatment	IVIG	None	None	None	N/A
Killed vaccines	Hepatitis B (no response before BMT)	Yes Normal specific Ab	Yes Normal specific Ab	Yes Normal specific Ab	None
Live-vaccines	No	Yes	Yes	No	None
Current age/follow up (months)	40	39	18	11	N/A
Outcome	Well and alive	Well and alive	Well and alive	Well and alive	Died at age 47 days

*Ab, antibodies; BMT, bone marrow transplantation; FTT, failure to thrive; Flu, Fluconazole; IVIG, intravenous immunoglobulins; N/A, not applicable; NEC, Necrotizing enterocolitis; TMP+SMX, Trimethoprim/sulfamethoxazole; TREC, T cell receptor excision circles*.

**Table 2:** Summary of clinical and immunological data of the patients.

	**Pt1**	**Pt2**	**Pt3**	**Pt4**	**Pt5**
Age^*^ (months)	0.5	0.5	0.5	0.5	1
**Lymphocyte subsets (× 10**^**3**^ **cells per microliter)**					
Lymphocytes	1.4 (3.4–7.6)	2.5 (3.4–7.6)	1.7 (3.4–7.6)	2 (3.6–8.9)	0.69 (3.6–8.9)
CD3^+^	0.16 (2.5–5.5)	0.99 (2.5–5.5)	0.49 (2.5–5.5)	0.44 (2.1–6.2)	0.35 (2.1–6.2)
CD4^+^	0.1 (1.6–4)	0.55 (1.6–4)	0.37 (1.6–4)	0.2 (1.3–3.4)	0.31 (1.3–3.4)
CD8^+^	0.06 (0.6–1.7)	0.59 (0.6–1.7)	0.2 (0.6–1.7)	0.4 (0.6–2)	0.07 (0.6–2)
CD20^+^	0.45 (0.3–2)	0.87 (0.3–2)	0.86 (0.3–2)	1.06 (0.7–2.6)	0.14 (0.7–2.6)
CD56 (%)	30 (6–30)	29 (6–30)	17 (6–30)	27 (6–30)	25 (6–30)
**T cell proliferation (cpm)**					
PHA 25 μg/ml	18%	50%	ND	ND	28%
**TREC (500 ng DNA; cutoff-400 copeis)**					
TREC	10	58	403	0	0
**TCR** **β** **repertoire**					
Number of skewed clones (±2 × SD)	2	5	2	1	6

*The values for healthy aged matched controls are presented in parenthesis; cpm, counts per minute; PHA, Phytohemagglutinin; TREC, T cell receptor excision circles*.

* *Age when blood test was performed*.

**Figure 1:**
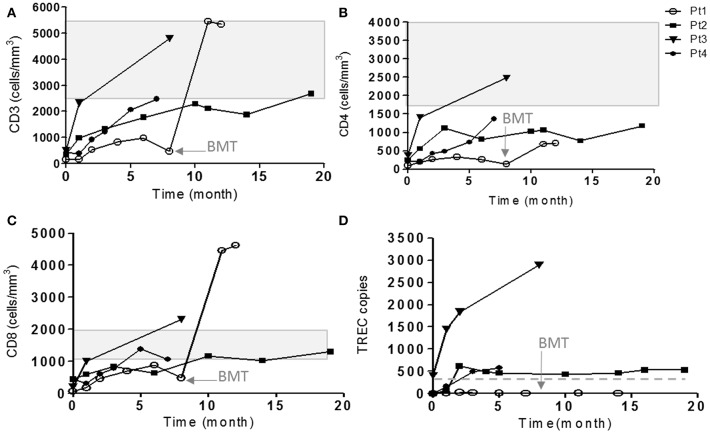
Immune evaluations of IL7Rα deficient patients. The cell surface markers of CD3 **(A)**, CD4 **(B)**, and CD8 **(C)** on patient's cells were detected during few months of follow-up by flow cytometry using immunofluorescent staining. The shaded area represents the age-dependent normal values of CD3, CD4, and CD8 according to Shearer et al. (35). **(D)** TRECs copy number were measured using genomic DNA isolated from patient's PBMCs in different time points. The horizontal solid line represents the cutoff values for normal TRECs. The age in which Pt1 underwent HSCT is indicated with an arrow (8 months).

During the first few months, the five patients developed distinct clinical disease course correlating with their follow-up immune evaluations (Figure 1). In order to better define the patients' immunodeficiency, TCR profile for patients was assessed using the classical TCR-Vβ flow cytometry assay. This revealed slightly abnormal pattern with some over and under expression of the different TCR Vβs for three patients (Table 2; Pt1, Pt3, and Pt4), but highly abnormal pattern in two patients (Table 2; Pt2 and Pt5).

Our patients eventually developed heterogeneous clinical presentations. Pt5 had severe scaly erythematous skin from birth and was initially suspected to have congenital Ichthyosis but diagnosed with *in-utero* Omenn syndrome (36). In addition to massive skin erythrodermia, Pt5 displayed other clinical features of Omenn syndrome including lymphadenopathy and organomegaly. The patient unfortunately succumbed to sepsis, due to complication by necrotizing enterocolitis (NEC). Pt1 developed CMV and adeno-viral infections and bacterial tracheitis (requiring PICU admission, intubation, and tracheostomy), presenting with significant clinical course of frequent hospital admissions and failure to thrive, even with isolation and antibiotic prophylaxis. At 8 months of age, she underwent successful non-conditioned HSCT from healthy matched sibling. Currently, she is well with 60% donor cells in her peripheral blood. Three patients (Pt2–Pt4) had a normal follow-up with no significant medical issues (Figure 1).

### Genetic Evaluation

The immune phenotype of the patients and the consanguinity of the patients' parents have pointed toward one of the autosomal recessive T^−^B^+^NK^+^ SCID diseases (e.g., IL7Rα, *CD3*δ, *CD45*). Whole exome sequencing (WES) was performed for four patients and their parents (Pt1–3 and Pt5). Sequencing yielded many variants in recessive analysis that were subsequently reduced to rare variants (Supplementary Table S1), by filtering out variants present in ≥0.01 of our in-house exomes (*n* = 1,235), and present with a minor allele frequency (MAF) ≥ 0.01 in either the 1,000 Genomes Project (1 KG; http://grch37.ensembl.org/index.html) or dbSNP 135 database or the NHLBI Exome Sequencing Project (ESP; http://evs.gs.washington.edu/EVS/; Supplementary Table S2). The only variant among these candidates that could significantly affect the immune system and was common to all patients was *IL7RA*, with a novel homozygous mutation in exon 2: n.c.120C>G; p.F40L (TTC to TTG) found in all patients. Six of seven computational programs, that were applied to assess changes in thermos-stability and function, predicted the p.F40L variant with severe pathogenesis. In addition, to determine whether there is a founder effect beyond the specific mutation, we investigated common SNPs surrounding the mutation. The SNP analysis for patients 1, 2, 3, and 5 and the parents of patient 1 and 2 revealed the presence of a common haplotype (Supplementary Table S3).

**Figure 2:**
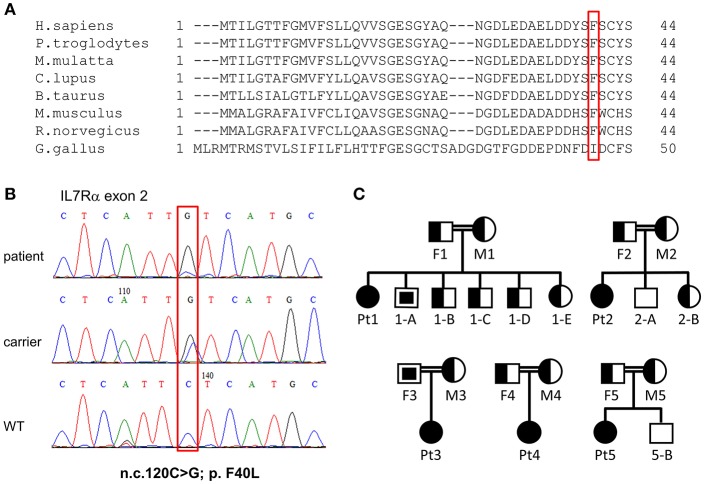
Validation of *IL7RA* mutation. **(A)** Multiple sequence alignment of the first 44 amino acids in human across different species, where the amino acid changed in our patients due to the missense mutation is boxed in red. **(B)** Sanger sequencing confirmed the presence of a missense mutation in the patients which fully segregated with the parents. The mutated nucleotide is boxed. **(C)** Pedigree diagram of the families of the affected patients. Filled shapes represent our patients, half-filled shapes represent the carriers for the mutation and non-filled shapes represent individuals without the mutation. The empty squares with the filled squares represent individual that carry homozygous mutations and they were not subjected to NBS (were born prior to the implementation of national screening for SCID). These individuals were discovered with homozygous mutation at the time of genetic validation of the affected patients.

Next, dideoxy Sanger sequencing confirmed the presence of this mutation in all the patients (including Pt4), which fully segregated with the parents (Pt1–Pt4; Figures 2B,C). For Pt5, the mutation was validated in the patient only. We repeated sanger sequencing few weeks post initial diagnosis, for Pt 2 and Pt3 who showed different clinical outcome, to exclude the possibility of reverse mutation. The analysis confirmed the presence of the mutation. To further exclude the remote possibility that the n.c.120C>G mutation is an ethnic SNP, WES database of similar ethnic background (Bedouin population, *n* = 360) was screened. No homozygous variant was found for the n.c.120C>G mutation and heterozygosity was found at a ratio of 1:120, which corresponds to homozygous genetic change with the frequency of 1/14,400 (6.9 ×10^−5^). This frequency correlate to the higher frequency of homozygous mutation associated with SCID in consanguine communities (37). In addition, this mutation was found to be highly conserved amino acid in a conserved region across different species (Figure 2A). During the genetic evaluation, a 3-year-old male sibling of Pt1 (1-A) and father of Pt3 (F3) were found to be also homozygote for the *IL7RA* mutation (Figure 2C). Although 1-A was considered clinically healthy, his immune workup was lower than normal during his clinical evaluation (Lymphocytes = 2.2; CD3 = 1.5; CD4 = 0.8; CD8 = 0.7; CD20 = 0.46 ×10^3^ cells per μl), including 352 TREC copies, which is close to the lower cut off value (400 TREC copies determined from peripheral blood). Furthermore, retrospective analysis of TREC copies in his dried blood spot obtained immediately after his birth was 23 copies, lower than the cut-off of 36 copies. This sibling was born before the newborn screening for SCID was implemented in Israel; therefore he was not identified at birth.

### Expression and Functional Assessment of IL7Rα Mutant Protein

**Figure 3:**
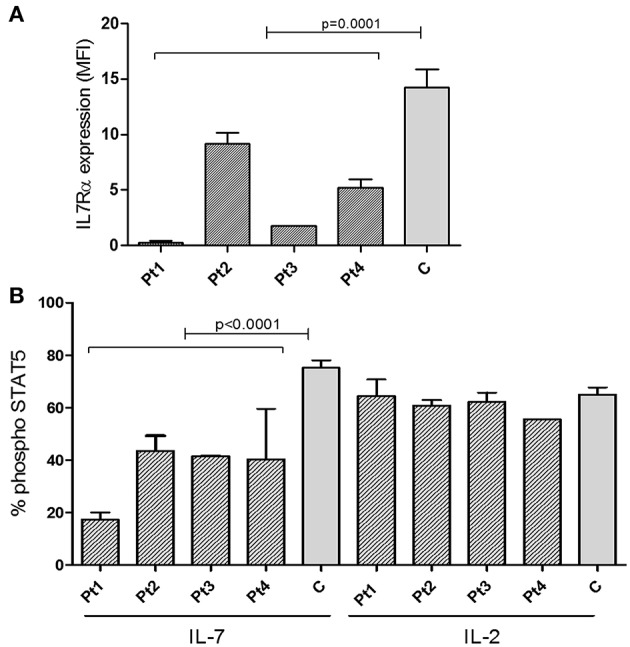
Expression of IL7Rα and Intracellular pSTAT5 phosphorylation determined by FACS. **(A)** Summary of Median Fluorescent Intensity (MFI) of IL7Rα positive CD3^+^ T cells from patients (*n* = 4, mean ± SE) and healthy controls (*n* = 9, mean ± SE). IL7Rα expression in all patients combined were significantly lower than the controls (*p* = 0.0001). **(B)** Measurement of phosphorylation of STAT5 in response to IL-7 and IL-2 stimulation in CD4^+^ T cells from IL7Rα deficient patients (*n* = 4, mean ± SE), and healthy controls (*n* = 10, means ± SE). Average data from 4 different experiments are shown. The phosphorylation of STAT5 in response to IL-7 were significantly lower in Pt1, Pt2, Pt3, and Pt4 compared with controls (Pt1, *p* < 0.0001; Pt2, *p* < 0.0001; Pt3, *p* = 0.0003; and Pt4, *p* = 0.0019). All statistical analyses were performed using unpaired one tailed *t*-tests.

The staining intensity of CD3^+^ T cells expressing IL7Rα in the blood samples of all patients showed significantly reduced intensity compared with the controls (Figure 3A and Supplementary Figure S2). Notably, in all patients, the IL-7 induced phosphoraylation of STAT5 (p-STAT5) upon stimulation with recombinant human IL-7 was reduced compared with controls (Figure 3B). As expected, activation with recombinant human IL-2 induced normal amounts p-STAT5 in patients' T-cells, compared to controls, emphasizing the specific defect in the IL-7-receptor-mediated signaling (Figure 3B). In addition, we analyzed the expression and function of IL7Rα in sibling 1-A which showed comparable results of the patients (Supplementary Figure S3). Thus we conclude that the IL7Rα expression is reduced and functionally impaired due to the n.c.120C>G mutation.

**Figure 4:**
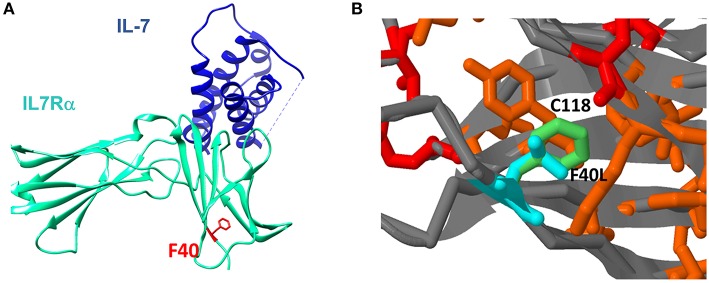
Structural context of F40L mutation in IL7Rα. **(A)** The mutated amino acid at position 40 is located close to the *N*-terminal site of the protein and it is spatially far from the IL-7 binding region. **(B)** Computational structure modeling of the interaction between F40 and C118. In red is the position with reported pathogenic variants according to ClinVar database. In orange are positions with somatic mutations in cancer according to the Cosmic database. In light green is the wild type Phenylalanine residue in position 40 and in light blue is the modeling of the mutant Leucine residue in position 40.

To further explore how the n.c.120C>G mutation affect the resulting protein, we used several computational tools. Programs such as Polyphen2, Sift2, and MutationTaster predict the effect on the protein function, concluded that the mutation is damaging/disease causing (Supplementary Table S4). Computational prediction tools based on protein structures (such as Imutant2, Cupsat, Maestro-web, and SDM) suggest that the p.F40L mutant reduces thermos-stability of the protein (Supplementary Table S4). We furthered explored the extracellular part of the IL7Rα, which is based on several x-ray crystallography structures and are available in the protein data bank [PDB (24)]. Looking at the structure of the IL7Rα with IL-7, it appears that position 40 is quite far (around 20 Å) from the protein-protein interface (Figure 4A) and therefore the mutation cannot directly affect the interaction with the IL-7. In addition, the F40 residue is structurally in close proximity to C118, where pathogenic variant was reported in several patients (18). Our structural model suggests that the p.F40L mutation dramatically reduces the contact area with C118 (Figure 4B), suggesting that local interactions and structural integrity in this region are important for the overall structure, stability and eventually proper function of IL7Rα.

### Skewed, Restricted, and Clonal Expansion of Patient's T Cell Receptor

**Figure 5:**
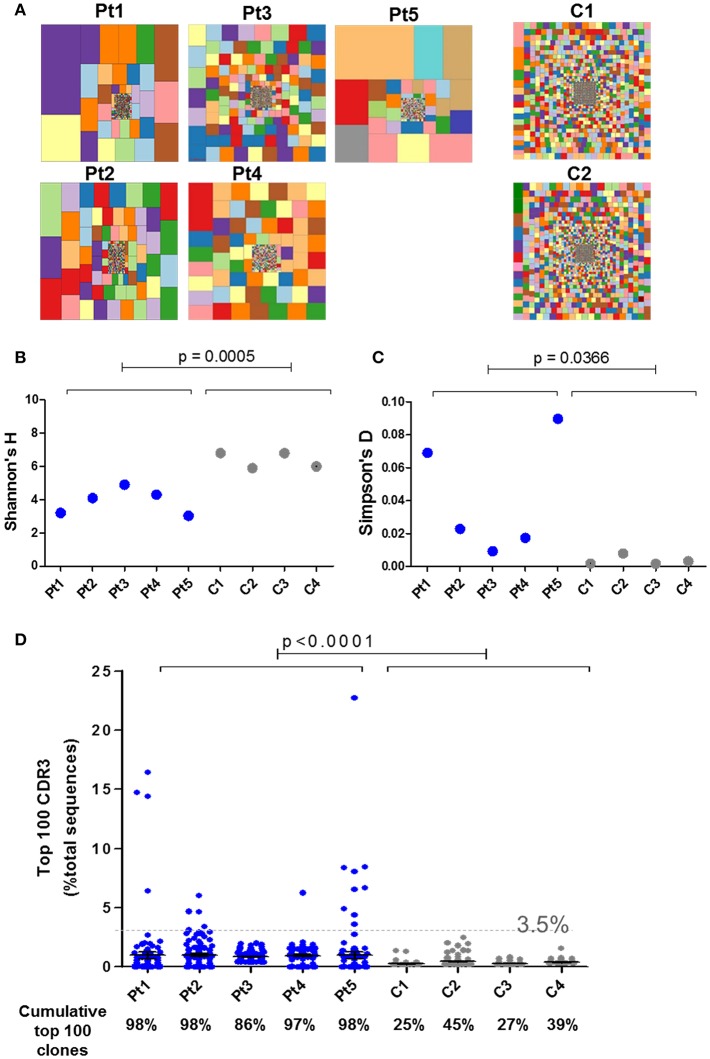
Immune repertoire determined by NGS for IL7Rα deficient patients. **(A)** Tree map representation of T cell receptor Gamma (*TRG*) repertoire in PBMCs samples from patients with IL7Rα deficiency and two healthy controls. Each square represents a unique V to J joining and the size of the square represents relative frequency within that sample. Two representative controls out of four is shown. Quantification of the diversity and unevenness of the *TRG* repertoire using the Shannon's H index of diversity **(B)** and the Simpson-D index of unevenness **(C)** in four healthy controls and in five patients with IL7Rα deficiency. There was no significant difference in the variance for Shannon's H index **(B)** however, the variance for the Simpson's D index **(C)** was greater in the patients (*F*-test; *p* = 0.0019). **(D)** Representation of the frequency of the top 100 most abundant TRG clones in IL7Rα deficient patients and healthy controls. Black horizontal lines representing average values. The dotted line (at 3.5%) represents the frequency of the highest values for the controls. The frequencies of the top 100 abundant clones were found to be significantly higher for each of the five patients compared to the controls (Pt1, *p* = 0.0086; Pt2, *p* < 0.0001; Pt3, *p* < 0.0001; Pt4, *p* < 0.0001, and Pt5, *p* = 0.0138). The cumulative percentage of top 100 clones are summarized at the bottom panel of **D**, where the values of the patients were significantly higher compared with controls (*p* < 0.0001). All statistical analyses were performed using unpaired one tailed *t*-tests and *F*-tests.

IL7 plays an essential role in the generation and maintenance of thymus-derived γδT cells (4, 5). Since IL7Rα-mediated signals are important for the TCR rearrangement of the γ locus (10, 38, 39), high-throughput immunosequencing of the *TRG* was performed on the blood samples from the patients and four healthy age-matched controls. There were significantly less unique and total sequence for all patients (Pt1–Pt5) at the time of diagnosis compared to the healthy controls (Supplementary Figure S4). Similarly, the hierarchical Treemaps demonstrate that the *TRG* repertoires of all the patients, were more restricted and clonally expanded than the age matched controls' repertoires (Figure 5A).

The Shannon's H Index estimates the diversity by taking into account both the number of total sequences and clonal size distribution in the overall repertoire. This quantitative measurement of diversity showed significantly lower values for patients compared with controls, reflecting restricted repertoire (Figure 5B). The Simpson's D Index measures the unevenness in the distribution of the size of clones within a repertoire. Higher values of Simpson's D were shown in patients compared with controls, demonstrating unequal distribution of individual clonotypes in the patients' *TRG* repertoire (Figure 5C). In order to determine the distribution of the diversity indices within the patient group, we analyzed for F-test to compare variances. Although the variance for the Shannon's H indices were similar between the patient and the control groups, the variance for the Simpson's D indices showed significant differences between the groups (Figure 5C). To further analyze the presence of clonal expansion, we calculated the frequency of the top 100 most abundant *TRG* CDR3 clones. All patients, except Pt3, showed expanded clones above 3.5% of total sequences, whereas the clones of the healthy controls were all below 3.5% (Figure 5D). Furthermore, the cumulative frequency of top 100 *TRG* CDR3 clones for the patients were significantly higher compared with the controls (Figure 5D; *p* < 0.0001). The variances of the frequencies of top 100 clones for all the patients were significantly different from the controls (Supplementary Figure S5; *F*-test; *p* < 0.0001). In addition, we analyzed the diversity of the *TRG* repertoire in sibling 1-A which showed more diverse repertoire than the patients, however the frequencies of top 100 clones were significantly higher compared to the controls (Supplementary Figure S6). Taken together, both graphical and quantitative measures of the repertoire diversity demonstrate that the *TRG* repertoire of the patients at the initial time of diagnosis are restricted and clonally expanded.

### Abnormalities in the CDR3 Region of TRG Repertoire

**Figure 6:**
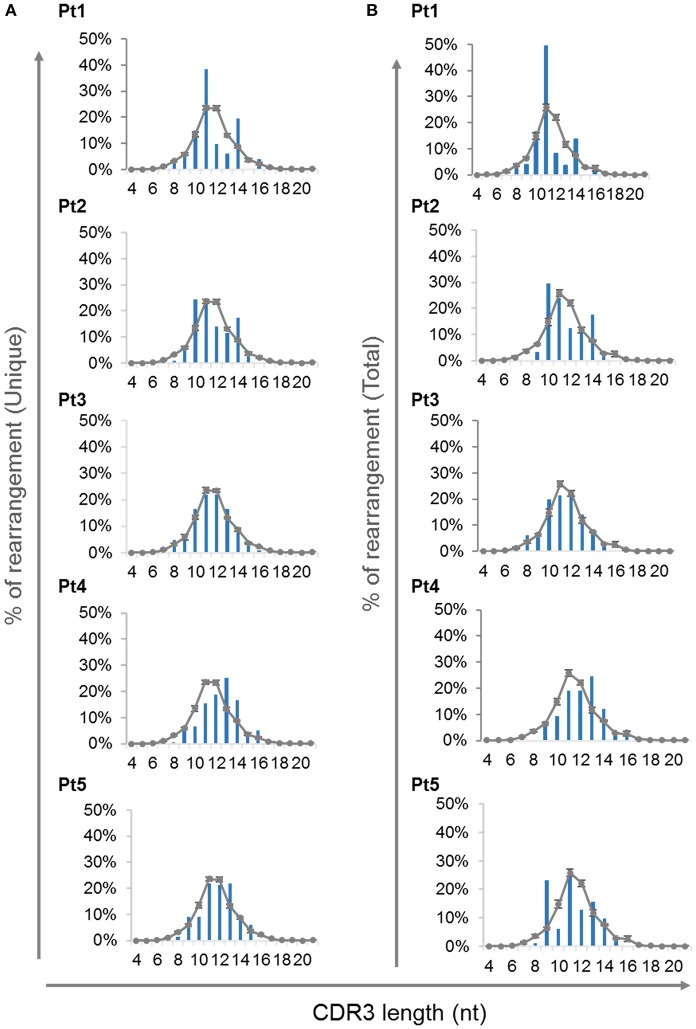
CDR3 length distribution of the *TRG* repertoire. The frequencies of various CDR3 length were calculated for each of the patients using the unique sequences **(A)** and total sequences **(B)** and were compared to the average of controls, depicted as a gray line (*n* = 4, average ± SE).

The complementarity-determining region 3 (CDR3) lie at the center of the antigen binding site and thus play an essential role in defining the specificity of the receptor. Abnormalities in the CDR3 length of TCR molecules have an important influence on the ability of the immune system to recognize variety of antigens (40). Analysis of CDR3 length distribution of unique TRG transcripts demonstrated skewing of the CDR3 length profile in all patients except Pt3 at the time of diagnosis (Figure 6A). Furthermore, the CDR3 length distribution of total sequences was similar to the unique sequences (Figure 6B).

**Figure 7:**
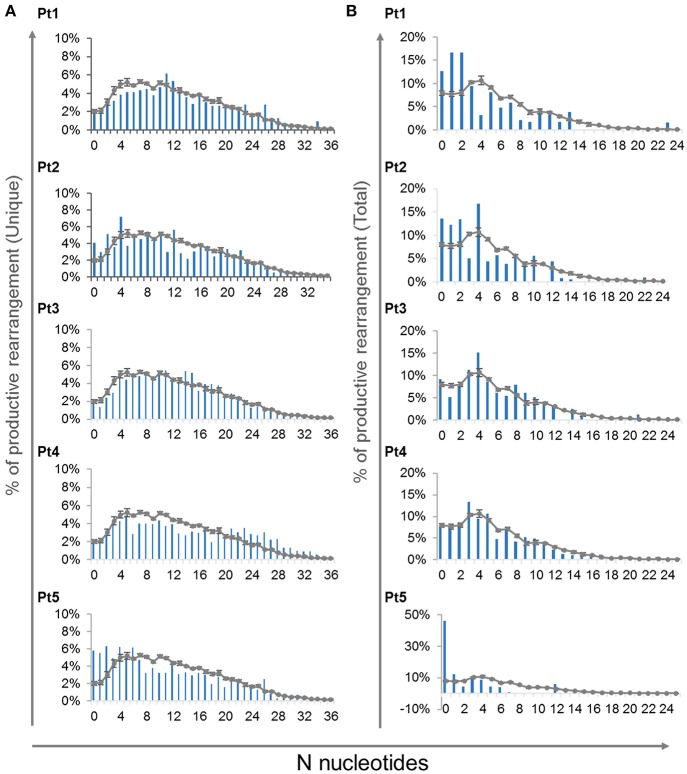
N-nucleotide additions in V-J junctions. Distribution of different number of N-nucleotide additions for the productive unique **(A)** and total **(B)** *TRG* rearrangements for each patient compared to controls (*n* = 4, average ± SE).

In order to determine the effect of the *IL7RA* mutation found in our patients on the junctional diversity during *TRG* rearrangement, we studied the N-nucleotide addition. It has been reported that IL7R signaling is important for N-nucleotide additions in postnatal B-cell precursors and patients with IL7Rα -deficiency contained fewer N-nucleotides addition in unproductive *IGH* gene rearrangements (41). In our patients, at the time of diagnosis, the average of N-nucleotides per junction in the unique and total for both unproductive and productive rearrangements were similar to the controls (Supplementary Figures S7A,B). Notably, the variance for the average N-nucleotide additions for the total sequences was significantly higher in the patients' compared with the controls' repertoire, both for the unproductive and productive rearrangements (Supplementary Figures S7A,B). This difference in the variance demonstrates that the expanded clones are most likely be with similar number of nucleotide additions in the CDR3 region of the controls' repertoire. However, the expanded clones of the patients' repertoire are with varying number of N-nucleotide additions. Lastly, we analyzed the distribution of N-nucleotide additions for the productive unique and total *TRG* rearrangements, which showed abnormal distribution of the N-nucleotide addition in the patients compared with the controls (Figure 7). Altogether these data imply that although the average CDR3 length and N-nucleotide additions did not show significant difference between the patients and controls, the junctional diversity of *TRG* is abnormal in our IL7Rα-deficient patients compared to controls.

## Discussion

Over the years, NBS using the TREC assay has revolutionized the early detection of SCID, with primary immunodeficiencies associated with T cell lymphopenia. This highly successful program enables us to identify patients with typical and leaky SCID. In many known SCID genes including *IL7RA*, leaky phenotypes have been reported (42–46). Importantly, patients with typical or leaky SCID phenotypes are prone to unfavorable outcome unless early HSCT is performed in order to restore their immunity (35). Here we present five patients who were identified by our national NBS as having SCID. We established their diagnosis by showing profoundly impaired T cell immune work up, pathogenic *IL7RA* mutation, dysfunctional IL-7 mediated signaling, and ability to engraft donor's cells without conditioning (Pt1), detectable maternal T cells in peripheral blood (Pt2), and severe congenital Omenn phenotype (Pt5). Patients with SCID, in particular, those who have been identified by NBS, undoubtedly benefit from earlier HSCT. However, there are barriers (i.e., familial, environmental, and others) that delay early intervention. While Pt1 clinically deteriorated and eventually underwent successful HSCT, Pt5 unfortunately succumbed to sepsis. Surprisingly, three patients displayed an unexpected immunological recovery without any significant clinical outcome. Yet, a prolonged and close follow-up is required to ensure the benign course of their disease.

In our current study, we have expanded our understanding of how this *IL7RA* mutation influences the development of immune cells by characterizing the *TRG* repertoire in patient's peripheral blood. It has been described that the effect of IL7R signals on recombination may be partially explained either by regulation of *RAG* gene expression or by chromatin accessibility in the *TRG* locus (38, 39). In our present study, the *TRG* repertoire of the IL7Rα deficient patients evaluated by high throughput immune-sequencing, revealed profound restriction and clonal expansions of the T cell receptor repertoires in both patients upon their initial diagnosis. Furthermore, skewing of the CDR3 length distribution was demonstrated in unique and total sequences from four out of five patients. In addition, the observation that the CDR3 length distribution was similar between the unique and total sequences indicates that these abnormalities can be attributed from the influence of *IL7RA* mutation on the primary rearrangement of the repertoire rather than environmental effects (i.e., infections and inflammations).

Recently, Rother et al. demonstrated that patients with genetic defects in *IL7RA* had lower levels of TdT expression and fewer N-nucleotides additions in the *IGH* gene rearrangements (41). Furthermore, the *TRG* rearrangement occurs during early T cell differentiation stages, where TdT expression is substantially increased (47). In our IL7Rα deficient patients, we found abnormal distribution of N-nucleotide addition in the *TRG* repertoire. Such an abnormality in the N-nucleotides addition has a direct effect on the junctional diversity. These data extend our understanding of the important role of IL7R signaling during *V(D)J* recombination and in creating the overall diversity of the *TRG* repertoire. Taken together, these data further support that IL7Rα plays a regulatory role in the shaping of the *TRG* repertoire.

The TRG repertoire was consistently restricted and expanded in all five patients even with the diverse clinical outcome of the patients. However, there are few characteristics of the TRG repertoire that may reflect the diverse consequences of IL7Rα deficiency. For instance, clonal expansions demonstrated by the Simpson's D and top 100 CDR3 show significant difference in the variances of the patients compared with controls. Furthermore, the variance of the average N-nucleotides in the total sequences was higher only for the patients. Altogether, these data from the TRG repertoire analysis suggest that the initial generation of the repertoire is affected due to the *IL7RA* mutation, whereas the degree of the clonal expansions and the characteristics of the expanded clones reflect the diverse clinical course that were presented by the patients.

For reasons yet to be explored, the mutation found in our patients have led to different clinical presentations. Furthermore, the presence of a sibling and a father with similar mutation suggests that this mutation may lead to leaky or hypomorphic phenotype, revealing incomplete penetrance. This phenomenon is well described in pathogenic mutations of the immune system, where same mutation in some genes may show variable numbers of immune cells, associated with various clinical and immunological phenotypes (48–51). Many hypotheses have been raised in order to explain this phenomenon. It was speculated that reduced penetrance and variable expressivity may be the factors that have an influence on the different clinical presentation due to genetic changes (52). Although these factors usually affect disorders that have an autosomal dominant pattern of inheritance, occasionally, they are seen in disorders with an autosomal recessive inheritance pattern (53), as in the presented case. These phenotypic differences are probably caused by a combination of genetic, epigenetic, and environmental factors, most of which have not been identified. In a recent review by Kammenga (54) it was suggested that the modifying genetic (such as genetic background, locus of the gene, combination of SNP with a specific mutation), epigenetic (histone modification) and environmental backgrounds (such as nutrition, underlying chronic disease) are as important as the “disease-causing” mutation itself. Furthermore, Copper et al. (55) have pointed toward allele dosage, differential allelic expression, copy number variation, sex, and age dependence as factors that influence the specific phenotype. Possibly, in depth study of minor modifier genetic changes in coding and non-coding areas using Whole Genome Sequencing and epigenetic studies by histone mapping will help to differentiate between variances in clinical presentations due to same mutation. Thus, from our study we can conclude that the *IL7RA* mutation leads to altered expression and defective function which negatively affects the *TRG* repertoire. The abovementioned modifying backgrounds (genetic, epigenetic, and environment) that are possibly influential shortly after birth, may affect the repertoire and consequently, the clinical presentation.

In summary, we are presenting a unique case where five unrelated patients share the same mutation in a rare genetic disorder of IL7Rα deficiency with divergent clinical course. Notwithstanding the limitations of patient number in this study, the patients were both identified by NBS, and at the time of diagnosis showed reduced TREC, low T cell count, impaired IL7R signaling, and profoundly restricted and skewed *TRG* repertoire, highly compatible with the diagnosis of SCID. Thus, together with the fact that no homozygosity was found in WES databases and no other pathogenic mutations were identified, suggests that the p.F40L IL7Rα mutant is indeed a disease causing mutation. However, other factors such as epigenetic, environmental, and variable expressivity may influence the ultimate clinical presentation.

## Data Availability

The datasets for this manuscript are not publicly available because it is available upon request. Requests to access the datasets should be directed to yuneeya4u@gmail.com.

## Ethics Statement

The Institutional Review Board (Sheba Medical Center, Tel HaShomer) approved this study and a written informed consent was obtained from their parents according to the ethical declaration of the Helsinki committee.

## Author Contributions

AL, AS, OBa, EE, EG-S, and ON performed research. OBi, TS, AB, SA, and RS collected data. AL, AS, EE, OBi, TS, AB, AHo, YL, and RS wrote the manuscript. AL, AS, OBa, EE, and ON analyzed data. AL, AS, ON, EE, YL, and RS interpreted data. AL, AHe, YL, and RS designed research.

### Conflict of Interest Statement

The authors declare that the research was conducted in the absence of any commercial or financial relationships that could be construed as a potential conflict of interest.

## Abbreviations

CDR3: Complementarity-Determining Region 3
JAK: Janus Kinase
MAF: minor allele frequency
NBS: New Born Screening
NGS: Next Generation Sequencing
PBMC: Peripheral Blood Mononuclear Cells
PID: Primary Immunodeficiency
Pt: Patient
SCID: Severe Combined Immunodeficiencies
STAT: Signal Transducer and Activation of Transcription
TREC: T cell Receptor Excision Circle
TRG: T cell receptor gamma
WES: Whole Exome Sequencing
FACS: Fluorescence Activated Cell Sorting.
