# Supplementary material for: Reduced Function and Diversity of T Cell Repertoire and Distinct Clinical Course in Patients With IL7RA Mutation
Source: Front Immunol. 2019 Jul 17;10:1672. doi: 10.3389/fimmu.2019.01672 (PMC6650764; doi:10.3389/fimmu.2019.01672)
Supplement: Supplementary file 1 [file Data_Sheet_1.docx]

**Supplementary Tables, Figure and Legend**

**Supplementary Figure S1.** **Measurement of non-self cells** **in *IL7RA* deficient patients.**

Percent chimerism over time was determined for Pt1 and Pt2.


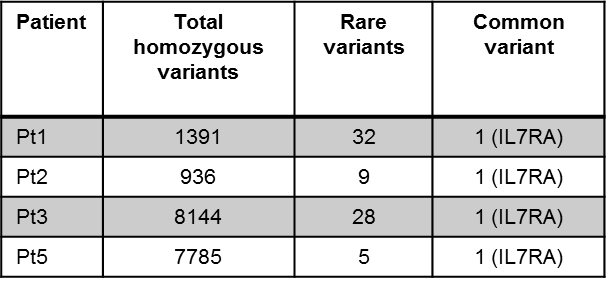


**Supplementary Table S1.** **Summary of the number of variants from WES analysis.**


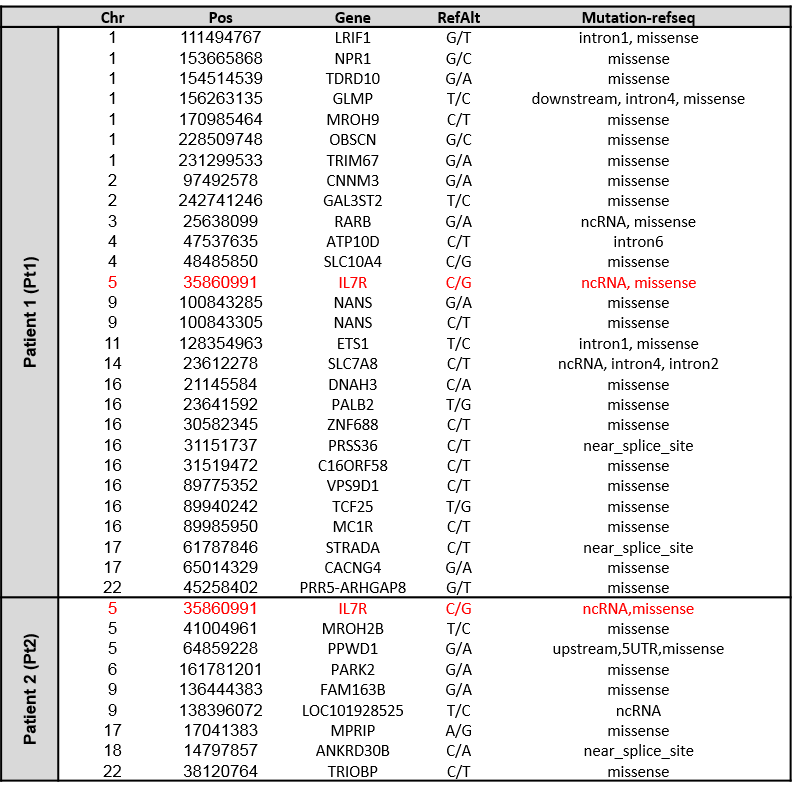


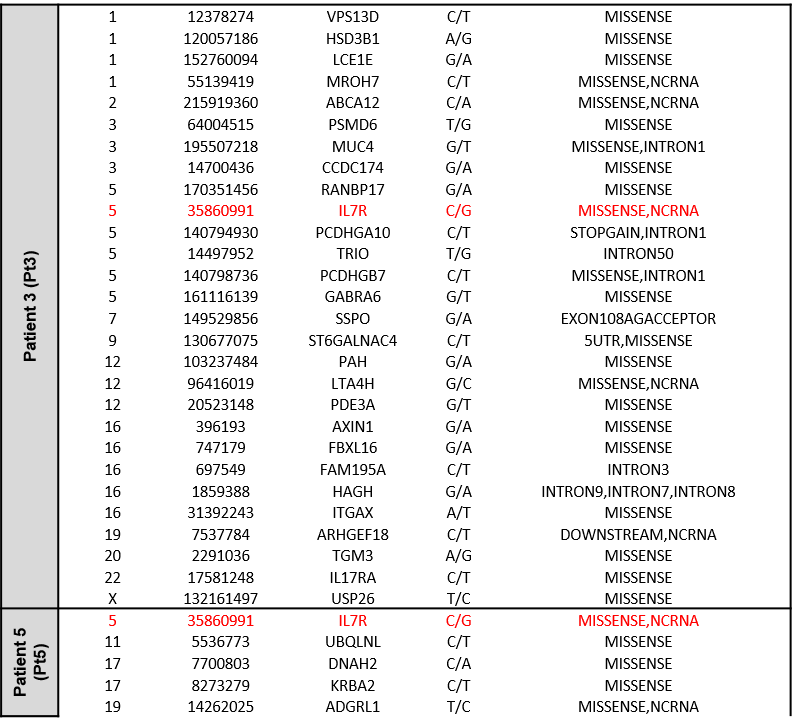


**Supplementary Table S2. Summary of the rare, homozygous variants from WES analysis.**

| SNP | **F1** | | **M1** | | **Pt1** | | **F2** | | **M2** | | **Pt2** | | **Pt3** | | **Pt5** | |
| --- | --- | --- | --- | --- | --- | --- | --- | --- | --- | --- | --- | --- | --- | --- | --- | --- |
| rs150841463 | A | G | A | G | G | G | A | G | A | G | G | G | A | A | A | G |
| rs56155801 | T | C | T | C | C | C | T | C | T | C | C | C | T | T | T | C |
| rs55647002 | C | A | C | A | A | A | C | A | C | A | A | A | C | C | C | A |
| rs6876028 | C | T | C | T | T | T | C | T | C | T | T | T | C | C | C | T |
| rs61737265 | C | T | C | T | T | T | C | T | C | T | T | T | C | C | C | T |
| rs16892150 | C | T | C | T | T | T | C | T | C | T | T | T | C | C | T | T |
| rs9282595 | C | T | C | T | T | T | C | T | C | T | T | T | C | C | T | T |
| rs61093907 | G | A | G | A | A | A | G | A | G | A | A | A | G | G | A | A |
| . | A | C | A | C | C | C | A | C | A | C | C | C | A | C | C | C |
| . | C | T | C | T | T | T | C | T | C | T | T | T | C | T | T | T |
| rs56053392 | T | G | T | G | G | G | T | G | T | G | G | G | G | G | G | G |
| rs6861184 | G | A | G | A | A | A | G | A | G | A | A | A | A | A | A | A |
| rs9292610 | G | C | G | C | C | C | G | C | G | C | C | C | C | C | C | C |
| rs7714297 | A | G | A | G | G | G | A | G | A | G | G | G | G | G | G | G |
| rs6875303 | A | G | A | G | G | G | A | G | A | G | G | G | G | G | G | G |
| rs73090222 | T | C | T | C | C | C | T | C | T | C | C | C | C | C | C | C |
| **IL7R** | **C** | **G** | **C** | **G** | **G** | **G** | **C** | **G** | **C** | **G** | **G** | **G** | **G** | **G** | **G** | **G** |
| rs11567764 | G | A | G | A | A | A | G | A | G | A | A | A | A | A | A | A |
| rs73076137 | G | A | G | A | A | A | G | A | G | A | A | A | A | A | A | A |
| rs111715633 | T | G | T | G | G | G | T | G | T | G | G | G | G | G | G | G |
| rs148290056 | G | A | G | A | A | A | G | A | G | A | A | A | A | A | A | A |
| rs377481973 | AC | A- | AC | A- | A- | A- | AC | A- | AC | A- | A- | A- | A- | A- | A- | A- |
| rs138582959 | G | A | G | A | A | A | G | A | G | A | A | A | A | A | A | A |
| rs2172777 | T | A | T | A | A | A | T | A | T | A | A | A | A | A | A | A |
| rs1874182 | G | A | G | A | A | A | G | A | G | A | A | A | A | A | A | A |
| rs12109571 | C | T | C | T | T | T | C | T | C | T | T | T | T | T | T | T |
| rs138067690 | TA | T- | TA | T- | T- | T- | TA | T- | TA | T- | T- | T- | T- | T- | T- | T- |
| rs73751622 | G | A | G | A | A | A | G | A | G | A | A | A | A | A | A | A |
| rs6891700 | C | T | C | T | T | T | C | T | C | T | T | T | T | T | T | T |
| rs10491422 | A | G | A | G | A | G | A | G | A | G | G | G | G | G | A | G |
| rs6884652 | T | C | T | C | T | C | T | C | T | C | C | C | C | C | T | C |
| rs57396930 | T | G | T | G | T | G | T | G | T | G | G | G | G | G | T | G |

**Supplementary Table S3. Summary of SNPs in the region of *IL7RA* mutation in patients and in the parents of Pt1 and Pt2.**


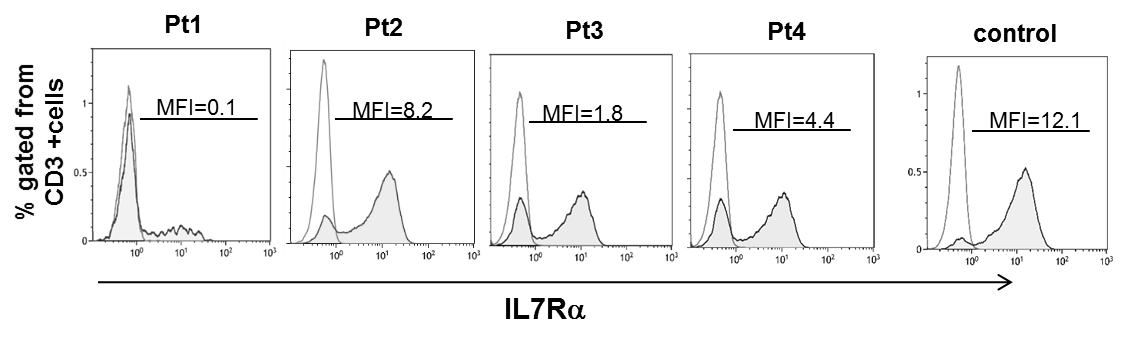


**Supplementary Figure S2. Expression of IL7Rα.** One representative experiment is shown, with Median Fluorescent Intensity (MFI).


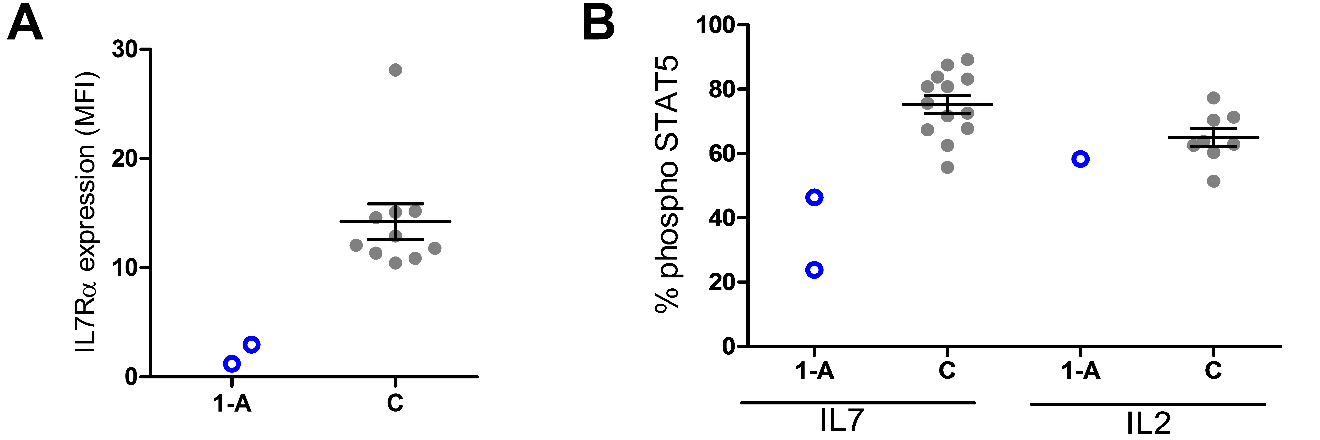


**Supplementary Figure S3. Expression of IL7Rα and Intracellular pSTAT5 phosphorylation determined by FACS for the sibling. A.** Summary of Median Fluorescent Intensity (MFI) of IL7Rα positive CD3^+^ T cells from sibling of Pt1, 1-A (n=2, mean ± SE) and healthy controls (n=9, mean ± SE). **B.** Measurement of phosphorylation of STAT5 in response to IL-7 and IL-2 stimulation in CD4^+^‏ T cells from the sibling with the same *IL7RA* mutation and healthy controls (n=10, means ± SE).


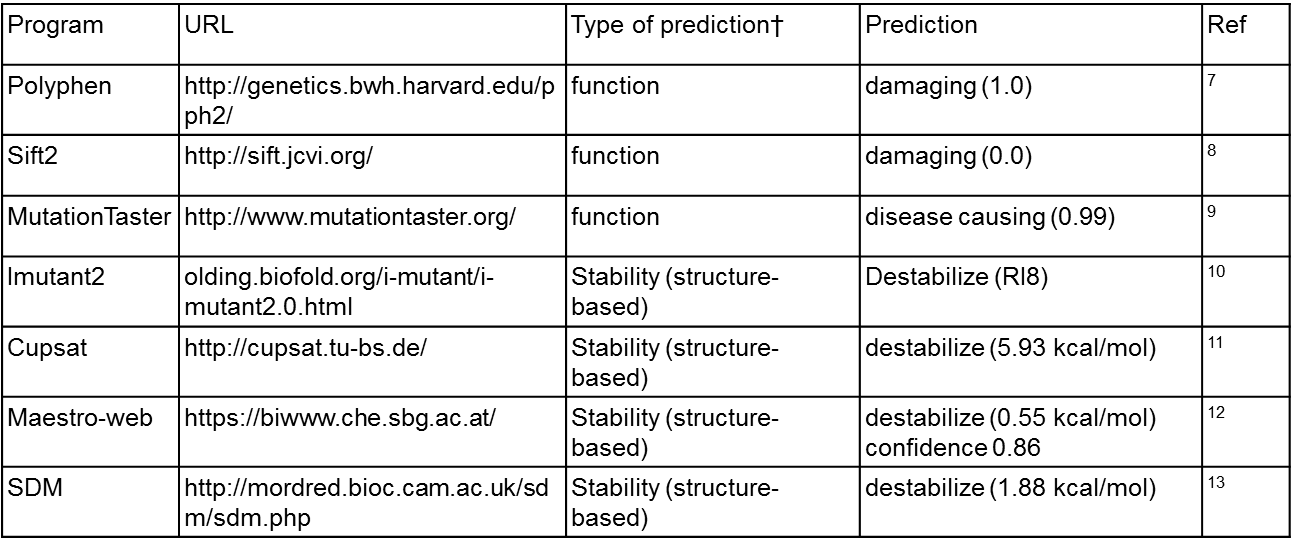


† All structural predictions are based on PDB file 3UP1 chain B

**Supplementary Table S4: computation prediction regarding the effect of the IL7R F40L mutations**


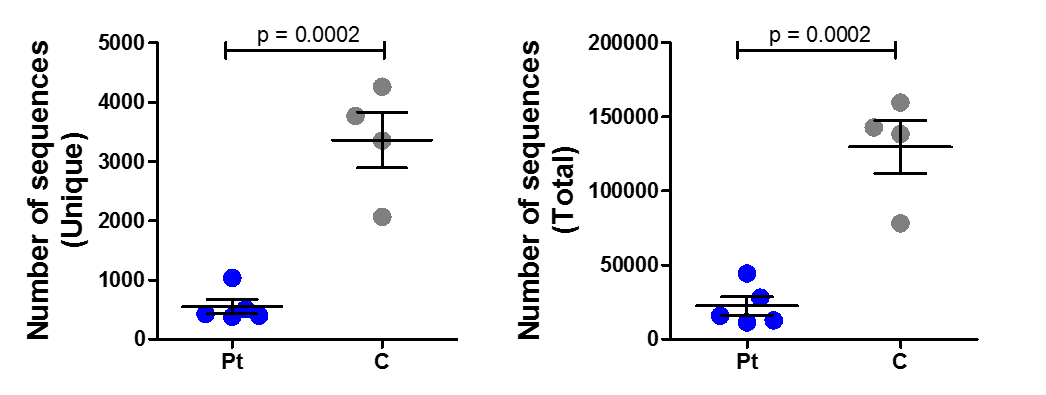


**Supplementary Figure S4. Number of Unique and Total sequences of TRG repertoire.** The graphs show significantly less unique and total sequences for the all the patients. All statistical analyses were performed using unpaired one tailed *t*-tests.

**Supplementary Figure S5. Analysis of the variance for the top 100 abundant clones.**  The graph represents the combined frequencies of top 100 clones from all five patients and from four controls. Statistical analyses were performed using unpaired one tailed F-tests.


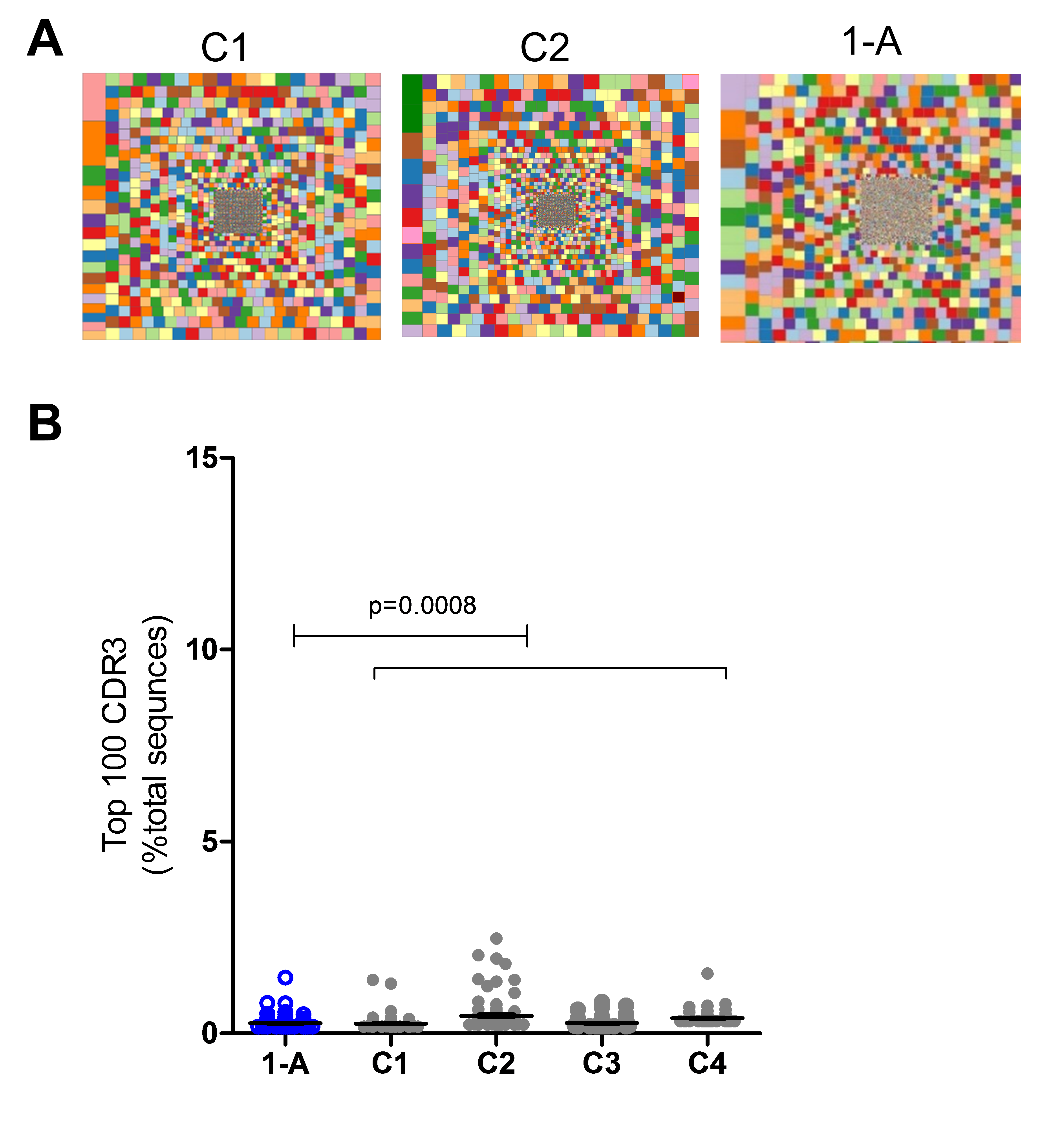


**Supplementary Figure S6. Immune repertoire determined by NGS for the sibling. A.** Tree map representation of T cell receptor Gamma (*TRG*) repertoire in PBMCs samples from the sibling with the *IL7RA* mutation and two healthy controls. Each square represents a unique V to J joining and the size of the square represents relative frequency within that sample. Two representative controls out of four is shown. **B.** Frequency of the top 100 most abundant TRG clones in the sibling with the *IL7RA* mutation and healthy controls. All statistical analyses were performed using unpaired one tailed *t*-tests.


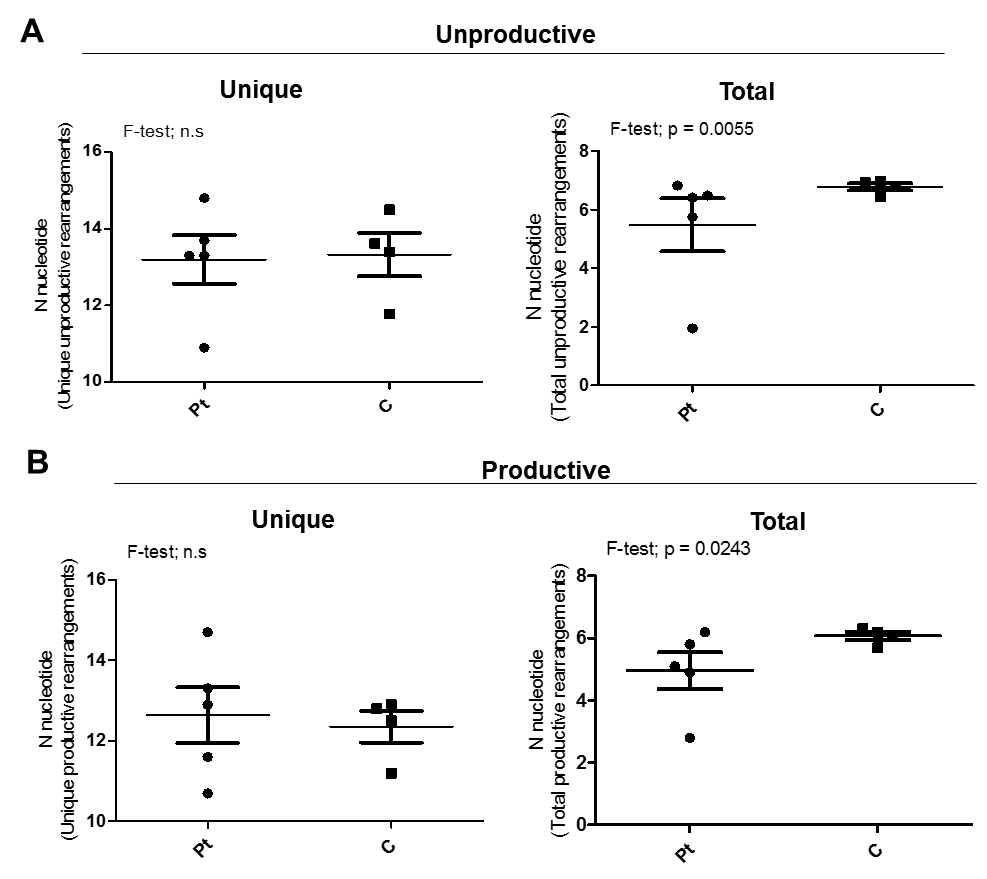


**Supplementary Figure S7. Average N nucleotide additions**. The average N nucleotide additions per sequence were calculated **A**. for the unproductive rearrangements using unique and total sequences and **B**. for the productive rearrangements using unique and total sequences. t-test showed no significant difference of the mean. The p values for the F-test are noted in the upper left corner of the graphs. All statistical analyses were performed using unpaired one tailed *t*-tests and F-tests.
